# Tristetraprolin is a novel regulator of BDNF

**DOI:** 10.1186/2193-1801-3-502

**Published:** 2014-09-06

**Authors:** Anmol Kumar, Kärt Varendi, Johan Peränen, Jaan-Olle Andressoo

**Affiliations:** Institute of Biotechnology, University of Helsinki, Helsinki, 00014 Finland

**Keywords:** Brain-derived neurotrophic factor, 3’ untranslated region, Tristetraprolin, C2C12 cells

## Abstract

Brain-derived neurotrophic factor (BDNF) regulates multiple biological processes ranging from central nervous system development and function to neuroinflammation and myogenic differentiation and repair. While coordination of BDNF levels is central in determining the biological outcome, mechanisms involved in controlling BDNF levels are not fully understood. Here we find that both short (BDNF-S) and long (BDNF-L) BDNF 3’UTR isoforms contain conserved adenylate- and uridylate rich elements (AREs) that may serve as binding sites for RNA-binding proteins (ARE-BPs). We demonstrate that ARE-BPs tristetraprolin (TTP) and its family members butyrate response factor 1 (BRF1) and 2 (BRF2) negatively regulate expression from both BDNF-S and BDNF-L containing transcripts in several cell-lines and that interaction between TTP and AU-rich region in proximal 5’ end of BDNF 3’UTR is direct. In line with the above, endogenous BDNF mRNA co-immunoprecipitates with endogenous TTP in differentiated mouse myoblast C2C12 cells and TTP overexpression destabilizes BDNF-S containing transcript. Finally, RNAi-mediated knock-down of TTP increases the levels of endogenous BDNF protein in C2C12 cells. Our findings uncover TTP as a novel regulator of BDNF assisting future studies in different physiological and pathological contexts.

**Electronic supplementary material:**

The online version of this article (doi:10.1186/2193-1801-3-502) contains supplementary material, which is available to authorized users.

## Introduction

BDNF is involved in a wide range of developmental, functional and pathological processes in the central nervous system (CNS) (Nagahara and Tuszynski 2011; Kirschenbaum and Goldman 1995; Cohen-Cory et al. 2010; Bamji et al. 2006; Nieto et al. 2013; Mu et al. 1999). Outside the CNS, processes regulated by BDNF include inflammation (Uchida et al. 2013; Lin et al. 2011; Gomes et al. 2013; Amoureux et al. 2008; Luhder et al. 2013), development of neuromuscular junctions (Je et al. 2012), muscle regeneration after injury (Clow and Jasmin 2010) and myogenic differentiation (Mousavi and Jasmin 2006).

Precise regulation of BDNF levels is critical in determining the biological outcome. Reduction of BDNF levels by 50% in BDNF knock-out heterozygous mice is associated with a range of phenotypes in the CNS (Lyons et al. 1999; Dluzen et al. 2002; Abidin et al. 2006; Abidin et al. 2008). On the other hand, a 2-fold elevation in endogenous BDNF by the suppression of miR-206, a direct negative regulator of BDNF levels, alleviates disease phenotype in a mouse model of Alzheimer’s disease (Lee et al. 2012). In muscle tissue, about 50% reduction in BDNF levels is believed to be required to allow myogenic differentiation (Mousavi and Jasmin 2006). However, despite the biological and potential clinical relevance, mechanisms controlling BDNF levels are not fully understood.

As a result of alternative polyadenylation, BDNF transcripts have either a long (BDNF-L, 2891 nt) or short (BDNF-S, 350 nt) 3’UTR (Timmusk et al. 1993). The ratio between transcripts containing BDNF-L and BDNF-S varies with BDNF-L levels ranging between 20-50% in different tissues and cells-lines (Timmusk et al. 1994; Miura et al. 2012). Alternative 3’UTR isoforms allow cells to differentially regulate expression from transcripts containing long or short 3’UTR.

Elements within the 3’UTR that control mRNA stability enable to adjust the expression of important regulatory proteins, including neurotrophic factors. Adenylate- and uridylate (AU)-rich elements (AREs) are typically 50–150 bp areas in the 3’UTR that serve as binding sites for *trans*-acting ARE binding proteins (ARE-BPs) which either stabilize or destabilize transcripts (Xu et al. 1997; Barreau et al. 2005). Although the exact consensus sequence of AREs is not in depth understood, AREs are often highlighted by high AU content and concomitant presence of AUUUA pentamers (Chen and Shyu 1995; Barreau et al. 2005). According to current estimations, approximately 8% of the human transcriptome contains AREs. However, relatively few AREs are experimentally verified as functional targets of ARE-BPs (Barreau et al. 2005; Bakheet et al. 2006; Barrett et al. 2012; Apponi et al. 2011; Gruber et al. 2011; Pascale and Govoni 2012). ARE-BPs tristetraprolin (TTP), butyrate response factor 1 (BRF1) and 2 (BRF2) form the TIS11/TTP family of ARE-BPs that target mRNAs for rapid degradation by binding to AREs (Hudson et al. 2004; Brooks and Blackshear 2013; Sanduja et al. 2011). TIS11/TTP family proteins are central regulators of the expression of inflammatory cytokines and several oncogenes (Hudson et al. 2004; Brooks and Blackshear 2013; Sanduja et al. 2011). Whether TIS11/TTP family could control the expression levels of neurotrophic factors, such as BDNF, has remained unknown.

In the current study, we used publicly available *in silico* tools to search for conserved AREs in BDNF-L and BDNF-S, and look for ARE-BPs co-expressed with BDNF in sites with known BDNF function. We find that ARE-BPs TTP, BRF1 and BRF2, but not ELAVL1 or ELAVL2, inhibit expression from luciferase reporters containing BDNF-L and BDNF-S, and that AUF1 has a mild inhibitory effect in the same assay. Using electrophoretic mobility shift assay (EMSA), we demonstrate a direct interaction between the 5’ region of BDNF-S and recombinant TTP protein and find that endogenous BDNF mRNA co-immunoprecipitates with TTP. In line with the above, over-expression of TTP destabilizes transcript containing BDNF-S. Finally, we show that siRNA-mediated down-regulation of TTP during myogenic differentiation of mouse myoblast C2C12 cells leads to increased BDNF protein expression. Altogether, our findings suggest that TTP is a new post-transcriptional regulator of BDNF expression.

## Materials and methods

### Cell culture

Human Embryonic Kidney 293 (HEK-293), Chinese Hamster Ovary (CHO), HeLa and C2C12 cells were cultured at 5% CO_2_ and 37°C in growth medium (GM) containing Dulbecco’s Modified Eagle Medium (DMEM, Invitrogen/Gibco) supplemented with 10% fetal bovine serum (FBS; SV30160, Thermo Fisher Scientific) and 100 μg/ml Normocin (InvivoGen). Cells were kept at sub-confluent density and split one day before plating for an experiment. C2C12 wells were seeded to 6-well plates pre-coated with 0.1% gelatin. Myogenic differentiation of C2C12 mouse skeletal myoblast cells was induced by replacing growth medium with differentiation medium (DM) containing DMEM supplemented with 2% horse serum (HS; B15-021, PAA) (Miura et al. 2012) and 100 μg/ml Normocin.

### Constructs and cloning

Gateway^®^ pcDNA™-DEST40 vectors encoding for ARE-binding proteins for expression in mammalian cells were obtained from Genome Biology Unit (Institute of Biotechnology, University of Helsinki, Finland). Long and short BDNF 3’UTR-s (BDNF-L and BDNF-S) were amplified from BAC clone RP24-149 F11 (RPCI-24: *Mus musculus* (C57BL/6 J male) BAC library; BACPAC Resources) using primers with *XbaI* sites (Additional file 1: Table S1), and cloned into *XbaI* site in pGL4.13 vector downstream Firefly luciferase gene (E6681,Promega) and in *XbaI* site in pGL4.73 vector downstream Renilla luciferase gene (E6911,Promega). Similarly, U1 and U2 fragments were amplified with primers containing *XbaI* sites (Additional file 1: Table S1) from BDNF long 3’ UTR sequence and cloned into pGL4.73 vector (E6911,Promega) and Bluescript KS + (Stratagene). All constructs were verified by sequencing. In addition, ARE-BP protein expression from various ARE-BP encoding Gateway^®^ pcDNA™-DEST40 vectors in HEK-293 cells was verified using Western blotting (Additional file 1: Figure S1a). We also assessed whether the C-terminal tag (V5 or His) encoded by pcDNA™-DEST40 vectors has an impact on RBP activity in luciferase assay and found no difference between the tested tagged and non-tagged ARE-BP activity (Additional file 1: Figure S1b and see below).

### Luciferase reporter assay

For luciferase reporter assay, cells were seeded to 96-well plates (pre-coated with 0.1% gelatin for HEK-293 cells), plating density per well was 15,000 (HEK-293 and CHO cells) and 10,000 (HeLa cells) in a volume of 100 μl one day before transfection. Reporter plasmids in 10:1 ratio (100 ng Luc-BDNF-L, Luc-BDNF-S, Luc-U1 and Luc-U2, if not indicated otherwise) and 10 ng either pGL4.73 [hRluc/SV40] or pGL4.13 [luc2/SV40]) as internal controls were co-transfected per well on 96 well plate to normalize the luciferase signal in dual luciferase assay. BDNF-L/BDNF-S transcript ratio in HEK-293 cells transfected with BDNF-L is 60/40, as assessed by QPCR analysis. Transfections were done according to standard protocol recommended for Lipofectamine 2000 (11668–019, Invitrogen). Growth medium was replaced with fresh cell culture medium after 3–4 hours after transfection. Luciferase assay was performed with Dual-Luciferase^®^ Reporter Assay System (E1960, Promega) as recommended by the manufacturer. Briefly, cells were lysed 24 hours after the transfection with Passive Lysis Buffer (E1960, Promega). Plates were either stored at -80°C or analyzed immediately with Dual-Luciferase Reporter Assay reagents. Results from each experiment were normalized to controls from the same experiment. Data for each figure panel was collected from a set of several experiments where constructs indicated on the figure were present in each experiment. We find that TTP suppressive effect on BDNF 3’UTR containing reporter gene expression is observed in all experiments, but the strength of inhibition on reporter gene expression varies up to about 1.7 fold between different sets of experiments. The reason for variance between different experimental sets is not known, but likely reflects normal variance in cell-culture experiments. Each experiment contained 3–4 replicates per construct/treatment and was repeated 2–8 times as specified in the figure legends.

### Recombinant TTP protein production

Human TTP open reading frame was cloned into a T7lac based vector containing a His-tag (Peranen et al. 1996). The vector was transformed into BL21 (DE3) cells (Novagen), and the protein was expressed in the presence of IPTG (isopropyl β-D-thiogalactoside) for 4 h at 24°C. The cells were lysed in buffer A (20 mM Tris HCl, pH 8.0, 0.5% Triton X-100, 10 mM b-mercaptoethanol, 0.4 mM PMSF) by sonication. Then, NaCl and imidazole were added to a final concentration of 0.5 and 0.02 M, respectively. After centrifugation (15,000 × *g* for 15 min at 4°C) the supernatant was passed through a 0.45 μm filter. The His-TTP protein was purified by the HisTrap kit according to the manufacturer (GE Healthcare). Buffer B (50 mM Tris–HCl (pH 8.0), 150 mM NaCl, 0.5 mM EDTA, 1 mM DTT) exchange was done by using a PD-10 column (GE Healthcare). His-tag was cleaved from His-TTP by AcTEV (Invitrogen). The His-tag and AcTEV were removed by application to a HiTrap Chelating column and TTP was collected from the flow-through. TTP was concentrated using an Amicon Ultra-4 filter device (Millipore). Aliquots of TTP were snap frozen in liquid nitrogen and stored at -80°C.

### Electrophoretic Mobility Shift Assay (EMSA)

Depending on the orientation of U1 and U2 fragments, T7 or T3 RNA polymerase was used to synthesize RNA probes *in vitro*. RNA 3' End Biotinylation Kit (20160, Thermo Scientific Pierce) was used to label RNA probe at the 3’ end according to manufacturer’s protocol. Biotin labeling efficiency was assessed by dot plot as recommended by the manufacturer. Biotin-labeled RNA probes were diluted (5-10x) based on labeling efficiency obtained by dot plot assay. Diluted RNA probes were incubated with different concentrations of purified TTP protein at room temperature for 30 minutes in 20 μl reaction mixture containing 10 mM HEPES (pH 7.3), 40 mM KCl, 3 mM MgCl_2_, 2 mM DTT, 5% glycerol and 2 mg/ml tRNA. The reaction was mixed with 1X REMSA loading buffer (Thermo Scientific Pierce) and run for 2 hours in 6% native polyacrylamide gel at 130 V. The bands were transferred to positively charged nylon membrane (Roche) and UV cross-linked at 120 mJ/cm^2^ for 1 min. The detection of biotin-labeled RNA probes was done using LightShift Chemiluminescent RNA EMSA Kit (20158, Thermo Scientific Pierce) according to manufacturer’s protocol.

### RNA isolations and quantitative real-time PCR

RNA from C2C12 cells was isolated using either TRI reagent (TR 118, Molecular Research Center Inc.) or Trizol (15596–018, Ambion) according to protocol provided by the manufacturers. RNA samples were treated with Turbo DNA-free DNase (AM1907, Invitrogen) to remove DNA. cDNA synthesis using random hexamer primers in a final volume of 20 μl was performed using Transcriptor First Stand cDNA synthesis kit (04896866001,Roche) as recommended by manufacturer. Quantitative real-time PCR (RT-PCR) was done with Lightcycler 480 real-time PCR system (Roche Diagnostics) using Lightcycler 480 SYBR Green I Master Mix. Three replicate wells were run for each sample. Primers used for RT-PCR are indicated in Additional file 1: Table S1.

### RNA interference

SMART pool siGENOME mouse Zfp36 (TTP) siRNA (M-041045-01-0005,Thermo Scientific) and siGENOME Non-Targeting siRNA Pools (D-001206-13, Thermo Scientific) were transfected to C2C12 cells 50–75 pmol each using Lipofectamine^®^ RNAiMAX Transfection Reagent (13778030, Invitrogen) as recommended by the manufacturer.

### Western blot

For detection of RBP expression in cell-lines, samples were run on 12% acrylamide gel for 1 hour and blotted to Hybond-ECL membrane (G1492720, GE Healthcare). The membrane was incubated in blocking solution (5% Non-fat milk, TBS, 0.1% Tween) for 30 minutes and incubated in anti-V5 Mouse Monoclonal Antibody (R960-25, Invitrogen) at 1:6000 dilution in the blocking solution for 50 minutes. The membrane was washed and incubated in polyclonal goat-anti-mouse HRP-conjugated secondary antibody (P0447, Dako) at 1:2000 dilution in the blocking solution for 50 minutes. Pierce ECL Western Blotting Substrate (32106, Thermo Scientific Pierce) was used for signal detection according to manufacturer’s instructions.

### Enzyme-linked immunosorbent assay (ELISA)

**Figure 1:**
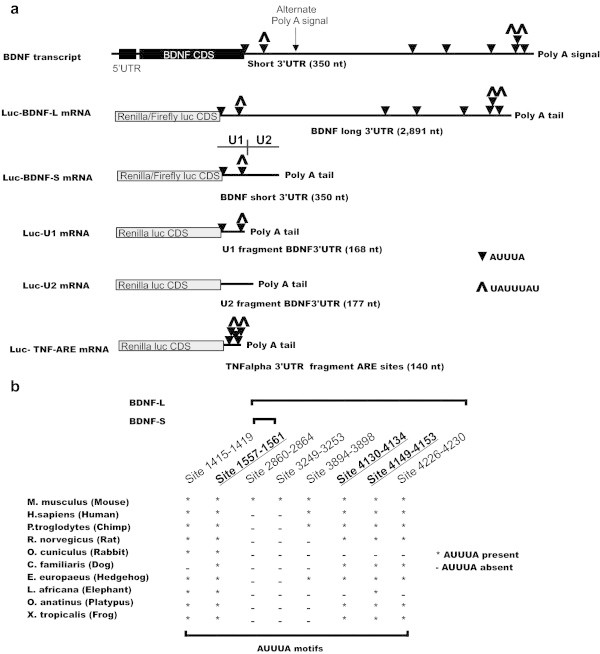
**AUUUA- motifs in mouse BDNF 3’ UTR and their conservation among vertebrates. a**. Schematic diagram of BDNF mRNA products with long (BDNF-L) and short (BDNF-S) 3’UTRs. AUUUA sequences, often serving as ARE-BP binding motifs within AU-rich elements (AREs) are indicated with black triangles, AREs UAUUUAU are indicated with up arrowheads, black box denotes BDNF coding sequence (Source: AREsite and AREscore). mRNA-s transcribed from luciferase reporter constructs (grey boxes) containing BDNF-L, BDNF-S, U1, U2 and ARE-rich fragment from TNFα 3’UTR. The positions of U1 and U2 (see below) are indicated above Luc-BDNF-S. **b**. Scheme depicting conservation of AUUUA sequences among vertebrates. UAUUUAU motifs are underlined and in bold.

Differentiation medium (DM) from C2C12 cells was collected at day 5 as described in the experimental plan in Figure 1a and centrifuged at 2000 rpm at +4°C for 2 minutes to remove cell debris. BDNF protein levels in the DM were measured using BDNF Emax^®^ ImmunoAssay System (G7611, Promega) according to manufacturer’s protocol. BDNF levels were normalized to total protein concentration using DC Protein Assay (500–0114, Bio-Rad) according to manufacturer’s recommendations.

### RNA immunoprecipitation (RNA-IP) assay

RNA-IP was performed as described previously (Ishmael et al. 2008). Briefly, differentiated C2C12 cells in six-well plate (CellStar) were lysed in 250 μl of polysome lysis buffer (100 mM KCL, 5 mM MgCl_2_, 10 mM HEPES (pH 7.0), 0.5% Nonidet P-40, 1 mM DTT, 100 U/ml RNaseOUT (Invitrogen), 0.2% vanadyl-ribonucleoside complex (New England Biolabs), Protease Inhibitor Cocktail (Roche)) and centrifuged at 12000 rpm for 15 minutes at 4°C. A/G agarose beads (Santa Cruz) were precoated with 10 μg of antibody against TTP or normal rabbit IgG (Santa Cruz). 100 μl of cell lysate was mixed with precoated A/G agarose beads in 900 μl of NT-2 buffer (50 mM Tris (pH 7.4), 150 mM NaCl, 1 mM MgCl_2,_ 0.5% Nonidet P-40) supplemented with 1 mM DTT, 100 U/ml RNaseOUT, 0.2% vanadyl-ribonucleoside complex and 20 mM EDTA and incubated for 2 hours at RT. Washed beads were incubated with NT-2 buffer supplemented with 0.1% SDS and 0.5 mg/ml Proteinase K at 55°C for 30 minutes. RNA was isolated and real time quantitative PCR was performed as described above.

### RNA turnover assay

HEK-293 cells were transfected as described above with Luc-BDNF-S, empty vector (pDEST 40) or TTP encoding plasmids. After 24 hours 1 μg/ml of Actinomycin D (A 1410, Sigma Aldrich) was added to the medium. Cells were lysed at indicated time points, RNA was extracted and semi-quantitative PCR was performed using primers indicated in Additional file 1: Table S1.

### Statistical analysis

For luciferase assay, data points from experiments within one experimental set (experimental set is defined as repeated experiments containing the same set of constructs) were averaged and used for statistical calculations. For other experiments, data was averaged from repeated experiments and used for statistical calculations. Error bars of graphs represent standard deviations (±SD). Data was analyzed with 2-tailed Student’s t-test assuming unequal variance. The level of significance was set at p < 0.01 unless otherwise indicated. Experiments were repeated 2–8 times with 2–4 replicates per experiment as indicated in specific figure legends.

## Results

### *In silico*prediction of ARE sites in the BDNF 3’UTR

Using publically available web tools AREsite (Gruber et al. 2011) (http://rna.tbi.univie.ac.at/cgi-bin/AREsite.cgi) and AREscore (Spasic et al. 2012) (http://arescore.dkfz.de/arescore.pl), we identified five conserved AUUUA motifs in the BDNF 3’UTR flanked by AU-rich sequences (Figure 1, Additional file 1: Figure S2). Two of the five AUUUA motifs were present in BDNF-S, while three AUUUA motifs were only present in BDNF-L (Figure 1, Additional file 1: Figure S2). Interestingly, three of the predicted AUUUA motifs are UAUUUAU (7-mer) sites, which may serve as high-affinity binding sites for TTP family ARE-BPs (Hudson et al. 2004; Brooks and Blackshear 2013) and one of the core 7-mer motifs is located in BDNF-S (Figure 1 Additional file 1: Figure S2).

### TTP family ARE binding proteins inhibit expression of a luciferase reporter containing BDNF-L and BDNF-S

**Figure 2:**
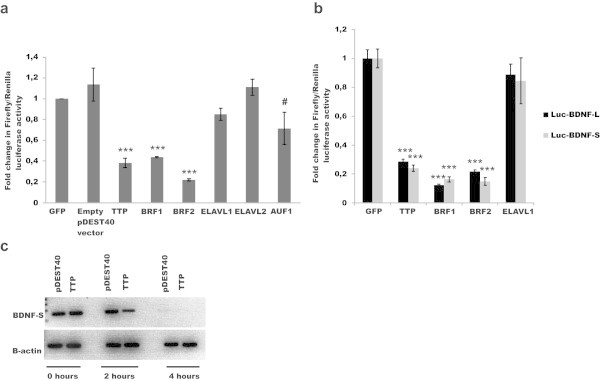
**Effects of ARE-BPs on expression from luciferase reporter containing BDNF-L or BDNF-S. a.** Effects of TTP, BRF1, BRF2, ELAVL1, ELAVL2 and AUF1 on the expression of luciferase reporter containing BDNF-L (Luc-BDNF-L) compared to Green Fluorescent Protein (GFP) and empty vector (pDEST40) as negative controls in HEK-293 cells, N = 2-8 experiments with 3–4 repeats per experiment, ***p < 0.001, #p < 0.035, Error bars indicate SD. **b**. Comparison of effects of TTP, BRF1 and BRF2 on Luc-BDNF-L and Luc-BDNF-S expression in HEK-293 cells revealed no statistically significant difference between BDNF-L and BDNF-S, N = 3-4, experiments with 4 repeats per experiment, ***p < 0.001. Error bars indicate SD. **c**. Effect of TTP expression on Luc-BDNF-S mRNA turn over in the HEK-293 cells upon Actinomycin D (1 μg/ml) treatment at the indicated time points, N = 2 experiments, a representative experiment is shown. BDNF-S and β-actin mRNA was detected with semi-quantitative PCR.

To analyze the biological importance of the *in silico* identified ARE motifs in BDNF 3’UTR, we cloned mouse BDNF-L and BDNF-S into luciferase reporter constructs (Figure 1a, see MM for details). Co-transfection of luciferase reporter constructs along with cDNAs encoding for ARE-BPs TTP, BRF1, BRF2, ELAVL1, ELAVL2 and AUF1 selected based on co-expression with BDNF in various tissues, (Additional file 1: Table S2) revealed that ARE binding proteins forming the TTP family - TTP, BRF1 and BRF2, but not ELAVL1 or ELAVL2, significantly inhibit expression of luciferase reporter containing BDNF-L in the human embryonic kidney 293 (HEK-293) cells, while AUF1 had a marginal effect (Figure 2a). Next we compared the effects of ARE-BP-s on BDNF-S and BDNF-L in the same reporter assay and found that the ability of TTP family members to suppress expression does not differ between luciferase reporter constructs containing BDNF-S and BDNF-L (Figure 2b). Then we studied the effect of TTP on the stability of a transcript containing BDNF-S upon transcriptional inhibition using actinomycin D. In line with results from luciferase assay, TTP overexpression destabilized BDNF-S containing transcript (Figure 2c). To test whether the observed inhibition by TTP family members is also observed in other cell-lines from different species we performed the luciferase assay in Chinese hamster ovary (CHO) cells and in Hela cells. TTP and BRF1 suppressed reporter gene expression in both cell-lines (Additional file 1: Figure S3a).

### TTP interacts with 5’ proximal end of BDNF-S without the requirement for AUUUA motif

**Figure 3:**
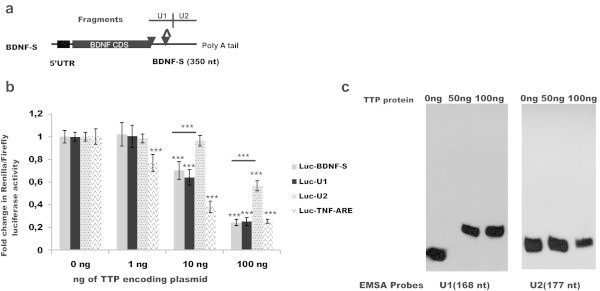
**TTP binds to the 5’ proximal fragment of BDNF 3’UTR. a**. Illustration showing the position of analyzed 3’UTR fragments U1 and U2 in BDNF-S, AREs are marked as on Figure 1a. **b**. Effects of 1–100 ng of plasmid/96-well encoding for TTP on reporter construct expression carrying BDNF-S, U1 and U2 in HEK-293 cells. TTP encoding plasmid has a moderate effect on U2 containing reporter construct expression only at the highest concentration (100 ng), while U1 is inhibited already at a 10 fold lower TTP encoding plasmid concentration (10 ng), suggesting different affinity of U1 and U2 towards TTP. A well-established target of inhibition by TTP, a 140 bp ARE-rich fragment from TNF-alpha 3’UTR (Luc-TNF-ARE) was included as a positive control, N = 2-3 experiments with 4 repeats per experiment. Error bars indicate SD, ***p < 0.001. **c**. Electrophoretic mobility shift assay showing band shift of RNA probe U1 with recombinant TTP protein (50 ng and 100 ng) and no band shift of RNA probe U2 under the same conditions. Experiment was repeated twice.

BDNF-S contains a proximal AUUUA site and a distal UAUUUAU site (Figure 3a). Both sites are conserved among vertebrates (Figure 1b) and may serve as binding site for ARE-BPs (Hudson et al. 2004; Brooks and Blackshear 2013). However, within the AU-rich region, (U)AUUUA(U) sequence may also be dispensable for RBP, including for TTP binding (Chen et al. 1994; Lopez de Silanes et al. 2004; Lopez de Silanes et al. 2005). To further characterize the suppressive effect of TTP on BDNF-S, we cloned the 5’ proximal end of the BDNF 3’UTR containing both conserved AUUUA site-s (designated as U1, Figure 3a) and the adjacent 3’UTR fragment (designated as U2, Figure 3a) into luciferase reporter construct as above. The overall AU-content of U1 and U2 fragments is comparable (Additional file 1: Figure S4). As a positive control for TTP inhibition in the luciferase reporter system we used a well-characterized target of inhibition by TTP, a 140 bp AU-rich 3’UTR fragment from TNF-alpha gene (TNF-ARE), which contains five overlapping UUAUUUAUU motifs identified as high-affinity binding sites for TTP (Hudson et al. 2004; Barreau et al. 2005). The overall AU-content of TNF-ARE was comparable to AU-rich regions of U1 and U2 of BDNF 3’UTR (Additional file 1: Figure S4). We found that luciferase reporter containing BDNF-S, 5’ proximal BDNF-S fragment U1 and TNF-ARE are significantly inhibited at lower TTP concentrations than U2, the distal BDNF-S fragment, on which TTP had moderate suppressive effect only at the highest concentration (Figure 3b). Repression by TTP was comparable between Luc-TNF-ARE, Luc-BDNF-S and Luc-U1 at the highest TTP concentration (Figure 3b). To assess whether similar repression is observed in cells derived from other species we repeated the experiment in CHO cells and observed a similar outcome (Additional file 1: Figure S3b). To study whether inhibition of U1 containing reporter by TTP can be attributed to a direct interaction between TTP and U1, we synthesized RNA probes corresponding to U1 and U2, produced recombinant TTP protein and performed electrophoretic mobility shift assay (EMSA). EMSA analysis showed a shift in the band representing U1 probe but no shift in the band representing U2 probe after addition of recombinant TTP protein (Figure 3c), suggesting a stronger interaction between TTP protein and U1 fragment of BDNF 3’ UTR. (U)AUUUA(U) elements within the AU-rich region may either be important or dispensable for binding by RBP-s (Chen et al. 1994; Lopez de Silanes et al. 2004; Lopez de Silanes et al. 2005). To discriminate between these two options, we studied the requirement of proximal AUUUA site, the distal UAUUUAU site and the necessity of both sites together in U1 (Figure 3a) for suppression by TTP. We generated constructs where the proximal AUUUA site (Luc-BDNFSmut1), distal UAUUUAU site (Luc-BDNFSmut2) and both (Luc-BDNFSmut1-2) AUUUA sequences were replaced with UAUAU. Changing either one or both AUUUA sequences to UAUAU did not affect the ability of TTP to inhibit BDNF-S (Additional file 1: Figure S3c), suggesting that BDNF-S falls into the class of 3’UTR-s where AUUUA elements are dispensable for RBP function (Chen et al. 1994; Lopez de Silanes et al. 2004; Lopez de Silanes et al. 2005).

### TTP interacts with BDNF mRNA and regulates endogenous BDNF levels in myogenic C2C12 cells

**Figure 4:**
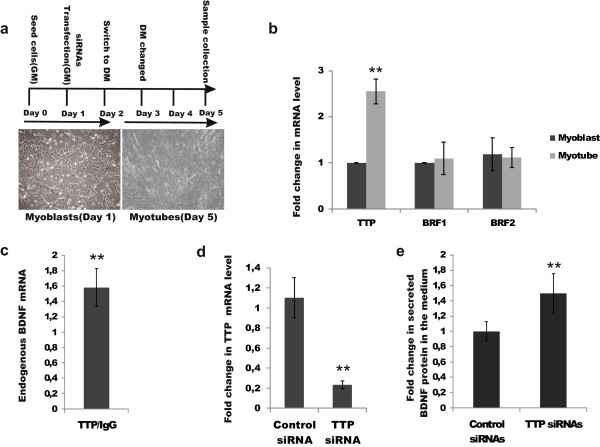
**TTP interacts with BDNF mRNA and regulates endogenous BDNF levels in myogenic C2C12 cells. a**. Outline of an experiment with C2C12 cells. Left: illustrative image of undifferentiated cells (myoblasts) at day 1; Right: illustrative image of differentiated cells (myotubes) at day 5. GM-growth medium, DM-differentiation medium. **b**. TTP but not BRF1 and BRF2 mRNA levels are upregulated in differentiating myotubes at day 5. **p < 0.01, N = 2-4, with 2–3 repeats per experiment. **c**. Endogenous BDNF mRNA co-immunoprecipitates with endogenous TTP protein in differentiating C2C12 cells, N = 2 experiments. **p = 0.01. **d**. Reduction in TTP mRNA levels after siRNA-mediated TTP knock-down at day 5, N = 4 experiments with 2–3 repeats per experiment, **p < 0.01. **e**. Increase in BDNF protein levels after siRNA-mediated TTP knock-down (c) in the cell-culture medium at day 5. N = 3 experiments with two repeats per experiment, **p < 0.01. Error bars indicate SD on all graphs.

TTP is acutely and transiently induced within 30 minutes following skeletal muscle injury in satellite cells, which give rise to myoblasts (Sachidanandan et al. 2002; Apponi et al. 2011). However, targets of TTP regulation upon muscle differentiation are unknown. On the other hand, BDNF is expressed in skeletal muscle satellite cells and down-regulation of BDNF levels is believed to be required to allow myogenic differentiation of myoblasts into myotubes (Mousavi and Jasmin 2006; Miura et al. 2012;). Our results suggest that TTP may suppress BDNF levels by directly interacting with its 3’UTR. To gain further insight into this, we first studied the expression levels of TTP, BRF1 and BRF2 in differentiating C2C12 cells, an established model of muscle differentiation (Burattini et al. 2004). Confirming and extending the earlier findings, we found that TTP, but not BRF1 or BRF2, is upregulated upon differentiation in C2C12 cells (Figure 4a-b). Next, we performed RNA-immunoprecipitation in differentiating C2C12 cells using antibodies against TTP and found that endogenous TTP co-immunoprecipitates with endogenous BDNF mRNA (Figure 4c). Finally, we tested the effect of RNAi knock-down of endogenous TTP (Figure 4d) on endogenous BDNF levels in C2C12 cells, and found concomitant upregulation of BDNF protein levels (Figure 4e).

## Discussion

Recently, 3’UTRs have emerged as an important site of gene expression regulation by binding of micro-RNAs (miRs) and RBPs. Long and conserved 3’UTRs that provide a binding platform for miRs and RBPs are especially common for regulatory genes, such as neurotrophic factors (Barrett et al. 2012). During the last two years, several miRs have been shown to directly interact with BDNF 3’UTR and regulate its expression(Miura et al. 2012; Lee et al. 2012; Varendi et al. 2014). However, how RNA binding proteins impact BDNF expression has largely remained unexplored. Microarray analysis of primary fibroblasts derived from TTP deficient mice revealed BDNF mRNA among ca 250 elevated mRNAs (Lai et al. 2006), suggesting that BDNF is either directly or indirectly regulated by TTP. Our results suggest that the suppressive effect of TTP on BDNF mRNA level is direct.

BDNF and TTP family members are co-expressed in many tissues, suggesting that the observed regulation may be involved in controlling a range of biological processes from CNS function to inflammation and muscle differentiation. That fine-tuning of BDNF levels via its 3’UTR has physiological and clinical importance is illustrated by a recent study by Lee *et al.*, where anti-miR based knock-down of miR-206, a negative regulator of BDNF, increased endogenous BDNF levels and alleviated Alzheimer’s disease in a mouse model (Lee et al. 2012).

So far only one ARE-BP, ELAVL4, is reported to impact BDNF mRNA stability by specifically stabilizing the BDNF-L but not the BDNF-S isoform (Allen et al. 2013). BDNF-L makes up 20-50% of BDNF transcripts in the CNS (Timmusk et al. 1994). We find that TTP binds to the 5’ proximal end (U1) of the BDNF 3’UTR and suppresses expression from both BDNF-S and BDNF-L to a comparable extent. This suggests that in BDNF 3’UTR, either U1 is the only binding platform for TTP, that binding of TTP distal to U1 has no additive effect on suppression, or that in BDNF-L only the distal binding site is accessible for TTP. Future studies will help to discriminate between these options.

TTP was discovered and has been characterized as an important regulator of inflammation (Sanduja et al. 2011; Taylor et al. 1996; Carballo et al. 1998; Brooks and Blackshear 2013). BDNF is the first neurotrophic factor identified as a target of TTP regulation. Interestingly, similar to TTP, BDNF regulates inflammatory processes including microglial activation and neuropathic pain (Uchida et al. 2013; Lin et al. 2011; Gomes et al. 2013; Amoureux et al. 2008), as well as neuroimmunological disease (Luhder et al. 2013). How TTP-mediated regulation of BDNF impacts the inflammatory processes remains to be explored.

Interestingly, as a rule, ARE-BP proteins share target mRNA-s. For an example GM-CSF, TNF-alpha and c-fos are shared targets of AUF1, ELAVL1 and TTP, see (Barreau et al. 2005) for review. We find that BDNF 3'UTR is directly regulated by TTP but the effect of AUF1 and ELAVL1 is either minor or absent. Therefore, further analysis of BDNF 3’UTR involving bioinformatics and laboratory techniques may enable to disclose which sequence/structure components in the BDNF 3’UTR underlie its specificity for TTP.

Taken together, co-expression of BDNF and TTP family members in various cell types and tissues suggests potential relevance of the interaction between TTP family and BDNF 3’UTR isoforms in different physiological and pathological processes. We hope our work facilitates future studies addressing the physiological and therapeutic potential of TTP/BDNF interaction in various organ systems.

## Electronic supplementary material

Additional file 1: Table S1: List of cloning and Q-PCR primers. **Table S2.** BDNF is co-expressed with ARE-BPs in various tissues with known BDNF function. **Figure S1.** **a**. Western blotting using antibodies against V5-tag showing that ARE-BP encoding constructs used in the study result in proteins with expected sizes. In addition, constructs used in this study were sequenced (see also MM). **b**. C-terminal tag (V5 or His) has no effect on TTP, ELAVL1 and ELVAL2 ability to modulate expression from reporter construct containing Luc-BDNF-L. N = 2 experiments with 4 repeats per experiment, ***p < 0.001. Error bars indicate SD. **Figure S2.** Mouse Bdnf 3’UTR sequence. Location of AUUUA motifs is indicated by red, UAUUUAU motifs are red in italics; the sequence of BDNF-S is bold and underlined. **Figure S3.** **a**. Similar to HEK293 cells (Figure 3), inhibitory effect of TTP and BRF1 on BDNF-L expression is observed in Hela cells and in Chinese hamster ovary cells (CHO). N = 1-2 experiments with 4 repeats per experiment, error bars indicate SD, ***p < 0.001, **p < 0.01. **b**. Similar to HEK293 cells (Figure 4b), low concentration of TTP encoding plasmid (0.5 ng/96-well plate well) has inhibitory effect on TNF-ARE and U1 but not on U2 in CHO cells. N = 3 experiments with 3–4 repeats per experiment, ***p < 0.001. **c**. Mutation in 5’, 3’ or both AUUUA sites present in BDNF-S has no effect on TTP-mediated inhibition on expression from Luc-BDNF-S reporter construct. N = 2 experiments with 3–4 repeats per experiment, ***p < 0.001. Error bars indicate SD on all graphs. **Figure S4.** AU percent in BDNF 3’ UTR fragments U1, U2 and in TNFα 3’UTR ARE fragment. (PDF 5 MB)
