# Supplementary material for: Tristetraprolin is a novel regulator of BDNF
Source: Springerplus. 2014 Sep 6;3:502. doi: 10.1186/2193-1801-3-502 (PMC4164675; doi:10.1186/2193-1801-3-502)

Kumar et al. Supplementary Table 1

List of cloning and Q-PCR primers

Primers for cloning	Forward Primer	Reverse primer
BDNF long 3' UTR-XbaI	5'-TATTCTAGAACTGACCATTAAAAAGGGGA AGATAGTGG-3'	5'-AAATCTAGATTACAATAGGCTTCTGAT GTGG-3'
BDNF short 3' UTR-XbaI	5'-GCATCTAGATGGATTTATGTTGTATAGATTATAT-3'	5'-CAGTCTAGAATCTGTTTTCTGAAAGAGGGAC-3'
U1 fragment-XbaI	5'-GCATCTAGATGGATTTATGTTGTATAGATTATAT-3'	5'-AAATCTAGAGGTCATGGATATGTCCAA TAAAT–3'
U2 fragment-XbaI	5'-CGC TCT AGA GGA AAC AGT CAT TTG CGC A-3'	5'-CAGTCTAGAATCTGTTTTCTGAAAGAGGGAC-3'
TNF ARE-XbaI	5'-TTTTCTAGAAGCCTTCCTCACAGAGCCAG-3'	5'-CCCTCTAGAGAAGACAGCTTCCCACAC TG-3'
Primers for Q-PCR	Forward Primer	Reverse primer
Mus musculus ZFP36/TTP	5'-TCTCTTCACCAAGGCCATTC-3'	5'-ATCGACTGGAGGCTCTCG-3'
Mus musculus ZFP36L1/BRF1	5'-GCACAAGCACAACTTTCCGT-3'	5'-AATTGCAAGCGTGGAGGTTG-3'
Mus musculus ZFP36L2/BRF2	5'-GCACAAGCACAACTTTCCGT-3'	5'-AATTGCAAGCGTGGAGGTTG-3'
Mus musculus actin, beta	5'-CCAGTTCGCCATGGATGAC-3'	5'-GAGCCGTTGTCGACGACC-3'
Mus musculus BDNF (CDS, transcript variants 1-4)	5'-TACCTGGATGCCGCAAACAT-3'	5'-GCTGTGACCCACTCGCTAAT-3'

Kumar et al.   Supplementary Figure 1

a.

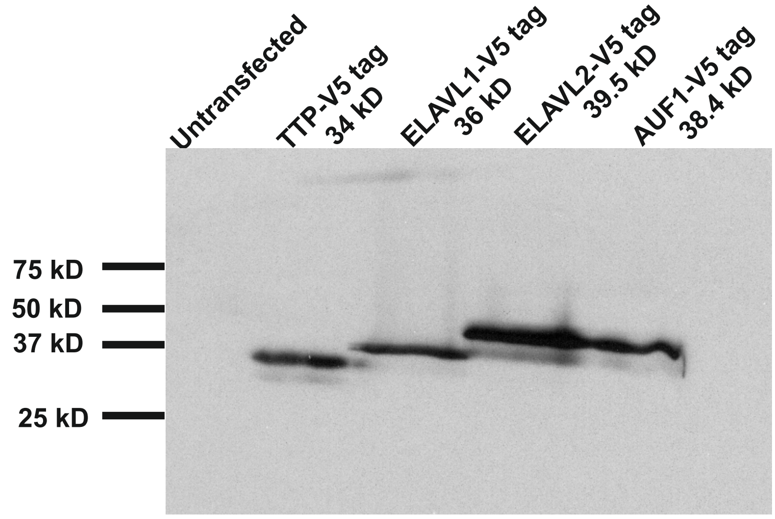

b.

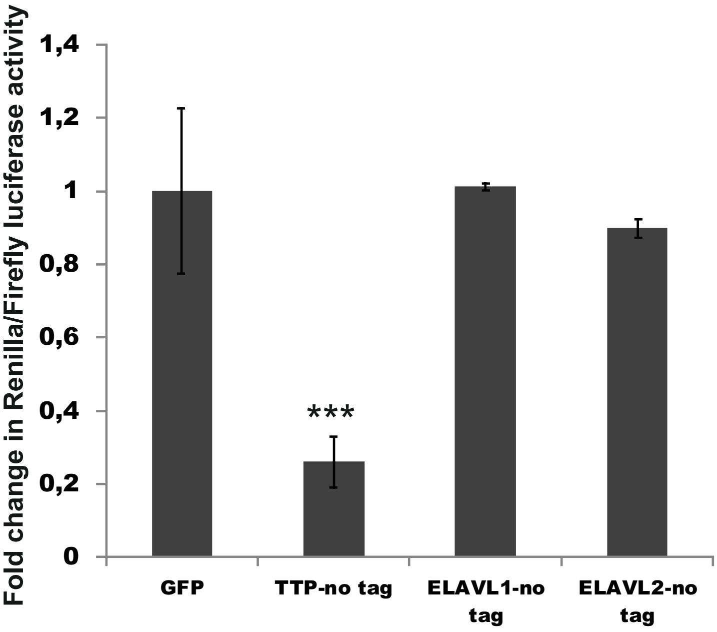

Kumar et al. Supplementary Figure 2

Mouse Bdnf 3’UTR sequence. Location of AUUUA motifs is indicated by red, UAUUUAU motifs are red in italics; the sequence of BDNF-S is bold and underlined.

1412 UGGAUUUAUGUUGUAUAGAUUAUAUUGAGACAAAAUUAUCUAUUUGUAUAUAUACAUAACAGGGUAAAUUAUUCAGUUAA

1492 GAAAAAAUAAUUUUAUGAACUGCAUGUAUAAAUGAAGUUUAUACAGUACAGUGGUUCUACAAUC*UAUUUAUU*GGACAUAU

1572 CCAUGACC*UGAAAGGAAACAGUCAUUUGCGCACAACUUUAAAAGUCUGCAUUACAUUCCUCGAUAAUGUUGUGGUUUGUU*

1652 GCCGUUGCCAAGAAUUGAAAACAAAAAGUUUAAAAAAAAAUAAUAAUAAAUUGCAUGCUGCUUUAAUUGUGAAUUGAUAAU

*1732 AAACUGUCCCUCUUUCAGAAAACAGAUUAAA*AAAACAAAAAACAAAAAAAAAAAAACAAAAAACAAAAACAAAAAUUGGA

1812 ACCAAAACAUUCCGUUUACAUUUUAGACACUAAGUAUCUUCGUUCUUGUUAGUACUCUGUUUUACUGCUUUCGACUUCUG

1892 AUAGCGUUGGAAUUAAAACAAUGUCAAGGUGCUGUUGUCAUUGCUUUACUGGCGUAAGGGACGGGGAAUGGGAGGGGUAG

1972 AUUUCUGUUUGUUUUGUGUUUUAUUUUGUUUGUUUGUUUGUUUUGUUUUUUAGUUCCACCCGGAGUAGGGAUGGAGAAAA

2052 UUUCUUCACUAUCCAUUCUGGUUGAUAAAGCGUUACAUUUGUAUGUUGUAAAGAUGUUUGCAAAAUCCAAUCAGAUGACU

2132 GGAAAACAAAUAAAAAUUAAGGCAACUGAAUAAAAUGCUCACACUCCACUGCCCAUGAUGUAUCUCCCUGGUCCCCCUCA

2212 GCUCACUCUUCUGGCAUGGGUCAGGGAAAAUUGCUUUUAUUGGAAAGACCAGCAUUUGUUCAAAGCAUACUCUUUCCCUC

2292 CCUCCUCCCAUUUUGGUCCCUUCUUUUUGUUUUGUUUUAAGAAAGAAAAUUAAGUUGCGCGCUUUAAAAUAUUUUACUAC

2372 UGCUACAAACAGAUGAACAAUGUGUGUCAUUUUAUGACACUCAUGGAAAACAGUGAUUUUUUUUUACCCUAAAGAAAAAC

2452 AAAUAAAAAUAACCCAAAAUAUUCUUUUUUUAAAAGGCAUAAAUAUUGGGUAAAUUGUAAUAUGGCCUAACAGUGUUUGC

2532 AGAUAAAAGUUAUUGUAUACACCCAGAUACUUAGAUAAGAGCAGGGAUCCACACUGCCAUUGAAAUAGGACUGAAUGGCC

2612 CUGCGGAGGCUAAGUGGAGCUGACAUACUAUUUCCUGGCAGUGCAGGAGGAAUUUCUGAGUGGCCAUCCUAAGGUCUAGG

2692 AUGGAGGUGGGGAAUGGUACUUGAGACAUUCCUAAAGGAAGGCUCGGAAGCACCCUUCAGAGCAGGCUCUGGAAUGAUGU

2772 GUCAAGUUGCUUAGGCCUUCUGCUUUAAGUGCCUACAUUACCUAACAGUGCUCAAGAGGUUCUCGAUUGGAGAACCACAC

2852 UCAAAUCCAUUUAUAGCCUCCAUCCCAUUUCUAAAUAAUUGUGUAUAAAGUUGGAUUAACCUGGAGCAACUUUGGAUCCA

2932 AAUAUGGCACAGCAAUAAUGAUAUUAAUGCAGCAUGAUGGGAAAUGUUUGCUGUGAAGAGAAUUGAUUUGCUUUGAGCUU

3012 AGACUUCAGGAAGCCUAGGUUUUUUAUUUUUUUAUUUUUGAGACAUUUUGGUAAAAGGAAAAAAAGAAAACAAACAAACA

3092 AACAAACAAAACCAGAAAAAGCAUCAAAACUCAGGCAGAAUGAGCAAUGUCUGAAAGGGCUAGAAAAACAAGACAUAGCA

3172 AGGUGCUUUCACUGUGAAAGAGACAAGAACACAGGAGGAAAUAUUGCUUCAGUGAAGAGCACAGACGGCUCCUGCCAAUU

3252 UAUUACAAGAGUCCCGUCUGUACUUUACCCUUUGGGGUUAGAAGUCAAGUUGGAAGCCUGAAUGAAUGGACCCAAUGAGA

3332 ACUAGUGUUAAGCCCAUUUCCCUAGUCAGGUUUUUUUCAAGCGUGAAUGUGUUAGUGGUUACUCUCCUGGGUUCCUGAGC

3412 AUCAGAAAAAAAAAAAAAAAGAGGCAAACAAUCGCUUCAUCUUAGGAGUGGAAAGGAAACAGAAGUGGACGUCCGCUGUG

3492 ACUCAGGGAGUGAAGAUACCAUCAGCAAAUAGUUUCUUUUUUGUUCAUUCGUUCCUUUCGAGUUAGCCUGUCUUUUGGAA

3572 UACCACUGAAUAUGCUGUUUUUGAAAGACUUCAUGUAGCAUAGAUUGUUUUGUGCCGUUUACCAAAUUAACCUUUGUCAU

3652 CGUUUUUUAACCUAUUCAGGAAUGCUUGGAAUAUCUGCUCUAUGUUAACUUUUUGCAGCUUCAUUCUGAGAGACAUUAGU

3732 CAAACAAACAAAAGGAUCCCCAUCACAAUCUUACAGUACUGCAAGGGCCAGGUCUGUUAAUCGGCUUCACAGGAGACAUC

3812 AGCAAUUGUGUGGUCAGUGGCUGGCUCUCUUACCCACUAAGAUACAUCAUAGCUACAUGUUGGUGGUUUAUGUUGACCUG

3892 AGAUUUAUUUGUUAAAAUCUCUUCUUCGUUUCUGUUCGUUCUGGUUCUGUUCUGUUCUGUUCUGUUCUGUUUUGGUUUUA

3972 AAGUCUUGCUGUGGUCUCUUGUUGGCAGAAAUGUUUUAUGCAUGGCAGCAGGCCUGUUGCUUUUUUAUAGUGAUUCCCAU

4052 UGAAACUGUAAGUAAAUGUCUGUGGCCUUGUUCUCUCUAUGGUAAAGAUAUUAUUCACCAUGUAAAACAAGAAAAAA*UAU*

4132 *UUAUU*GUAUUUUAGUA*UAUUUAU*AUAAUUAUGUUAUUGAAAAAAUUGGCAUUAAAACUUAACCACAUCAGAAGCCUAUUG

4212 UAAAUACAGGUUCUAUUUAAGUGUACCAAUUAACAUAUAAUAUAUGUUUUAAAUAUAGAAUUUUUAAUGUUUUUAAAUAU

4292 AUUUUCAAAGU

Kumar et al. Supplementary Table 2
BDNF is co-expressed with ARE-BP-s in various tissues with known BDNF function.

mRNA	Brain*	Skeletal muscle	Heart**	Monocytes
BDNF	Yes	Yes	Yes	Yes
TTP	Yes	Yes	Yes	Yes
BRF1	Yes	Yes	Yes	Yes
BRF2	Yes	Yes	Yes	Yes
ELAVL1	Yes	Yes	Yes	Yes
ELAVL2	Yes	Yes	Yes	Yes
AUF1	Yes	Yes	Yes	Yes

* BDNF is co-expressed with TTP, BRF1, BRF2, ELAVL1, ELAVL2 and AUF1 in prefrontal cortex, cingulate cortex, parietal lobe, temporal lobe, occipital lobe, ciliary ganglion, cerebellar penducles, globus pallidus, olfactory bulb, thalamus, hypothalamus, subthalamic nucleus, caudate nucleus, amygdala, pons
** BDNF is co-expressed with TTP,BRF1,BRF2,ELAVL1,ELAVL2 and AUF1 in heart and in primary human cardiac myocytes

SOURCE: www.Genecards. org

Kumar et al. Supplementary Figure 3

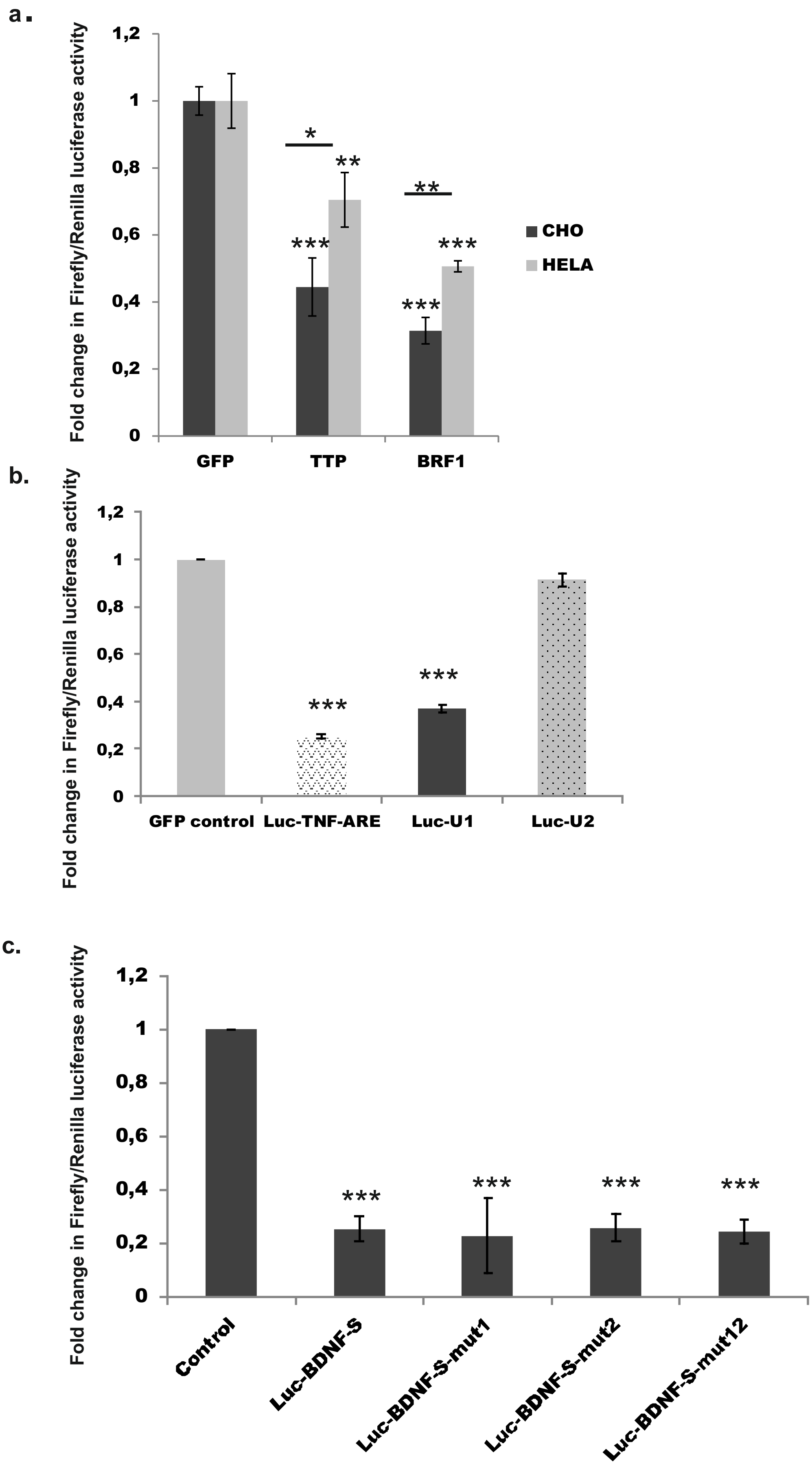

Kumar et. al. Supplementary Figure 4

U1

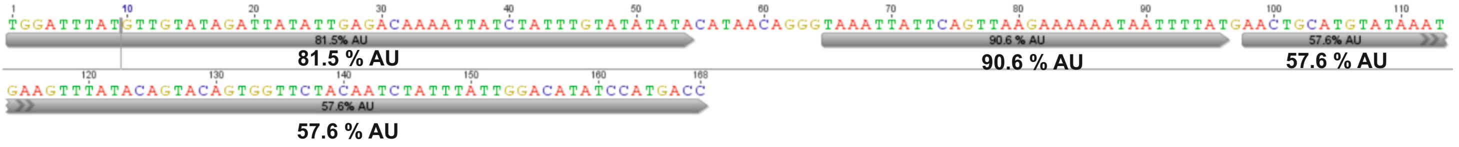

U2

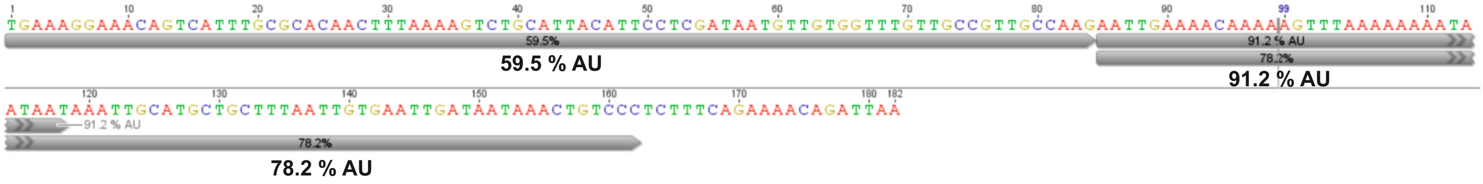

TNF alpha ARE

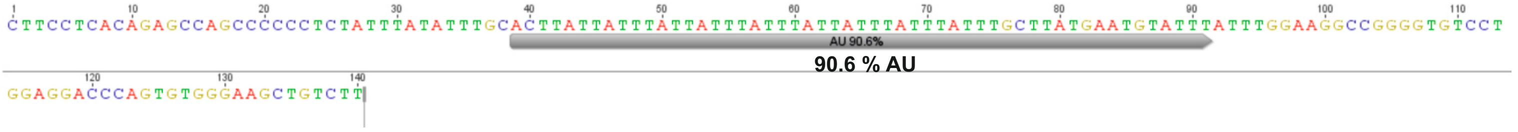

Supplement: Supplementary file 1 — Additional file 1: Table S1: List of cloning and Q-PCR primers. Table S2. BDNF is co-expressed with ARE-BPs in various tissues with known BDNF function. Figure S1. a. Western blotting using antibodies against V5-tag showing that ARE-BP encoding constructs used in the study result in proteins with expected sizes. In addition, constructs used in this study were sequenced (see also MM). b. C-terminal tag (V5 or His) has no effect on TTP, ELAVL1 and ELVAL2 ability to modulate expression from reporter construct containing Luc-BDNF-L. N = 2 experiments with 4 repeats per experiment, ***p < 0.001. Error bars indicate SD. Figure S2. Mouse Bdnf 3’UTR sequence. Location of AUUUA motifs is indicated by red, UAUUUAU motifs are red in italics; the sequence of BDNF-S is bold and underlined. Figure S3. a. Similar to HEK293 cells (Figure 3), inhibitory effect of TTP and BRF1 on BDNF-L expression is observed in Hela cells and in Chinese hamster ovary cells (CHO). N = 1-2 experiments with 4 repeats per experiment, error bars indicate SD, ***p < 0.001, **p < 0.01. b. Similar to HEK293 cells (Figure 4b), low concentration of TTP encoding plasmid (0.5 ng/96-well plate well) has inhibitory effect on TNF-ARE and U1 but not on U2 in CHO cells. N = 3 experiments with 3–4 repeats per experiment, ***p < 0.001. c. Mutation in 5’, 3’ or both AUUUA sites present in BDNF-S has no effect on TTP-mediated inhibition on expression from Luc-BDNF-S reporter construct. N = 2 experiments with 3–4 repeats per experiment, ***p < 0.001. Error bars indicate SD on all graphs. Figure S4. AU percent in BDNF 3’ UTR fragments U1, U2 and in TNFα 3’UTR ARE fragment. (PDF 5 MB) [file 40064_2014_1205_MOESM1_ESM.pdf]
